# Knowledge, Perceptions, and Practices of Skin‐Lightening Products Among Female Medical Students: A Cross‐Sectional Study

**DOI:** 10.1111/jocd.70380

**Published:** 2025-08-04

**Authors:** Hanadi Qeyam, Rula Al‐Shami, Ahmed Al‐Rusan, Mohannad Alnaimat

**Affiliations:** ^1^ Department of Dermatology Faculty of Medicine, Jordan University of Science and Technology Irbid Jordan; ^2^ Faculty of Medicine Jordan University of Science and Technology Irbid Jordan

**Keywords:** hydroquinone, skin tone, skin‐lightening products, vitamin C

## Abstract

**Objective:**

The use of skin‐lightening products (SLPs) is a widespread phenomenon influenced by cultural, social, and aesthetic perceptions. However, the awareness of their potential dermatological and systemic risks remains inadequate, particularly among young women, including medical students. This study aims to assess the knowledge, perceptions, and practices regarding SLPs among female medical students.

**Methods:**

A cross‐sectional survey was conducted among 305 female medical students at Jordanian universities. Data on participants' perception of skin color tone, knowledge of active ingredients, and the risks associated with the use of SLPs were collected and analyzed, in addition to exploring the prevalence and the pattern of the use of these products among them.

**Results:**

Participants exhibited good knowledge regarding SLPs, although their perceptions of lighter skin tones varied. 40.7% of participants reported using SLPs, with the majority having Fitzpatrick skin type 3. Vitamin C (72.4%) and niacinamide (38.3%) were the most frequently used active ingredients. The main reasons for SLP use were to treat facial hyperpigmentation (35.5%) and for general skin tone lightening (30.6%). Side effects included acne (30.3%), skin redness (29.4%), and color changes (16.8%). While 34.7% of users were satisfied with the results, 37.1% reported fractional improvement, and 28.2% were disgruntled. Only 43.5% consulted a physician or dermatologist before use, with many depending on social media and friends.

**Conclusion:**

The findings highlight a gap between awareness and practice, suggesting that social and cultural factors significantly influence behavior, potentially outweighing medical knowledge. This demonstrates the need for targeted educational interventions that address societal beauty norms, misinformation, and psychological dependence, as well as strengthening regulatory measures and promoting safe dermatological alternatives to reduce unsafe skin‐lightening practices among future healthcare professionals.

## 1 Introduction

In recent years, the way people perceive beauty has shifted significantly, influenced by media, cultural trends, technological advancements, and changing fashion ideals. However, despite these evolving standards, the demand for skin‐lightening products (SLPs) has continued to grow, especially in regions like Africa and Asia. This trend is highlighted by the rise in global internet searches for skin whitening treatments, indicating widespread interest in these practices [1, 2]. Women use SLPs for various reasons, primarily due to cultural pressure and the belief that lighter skin signifies beauty, success, and higher social status in certain regions. Additionally, SLPs are used for medical conditions such as melasma, lentigines, and post‐inflammatory hyperpigmentation [3, 4].

Multiple products have been introduced as whitening agents, including over‐the‐counter creams, herbals, and prescribed treatments; most of them contain common ingredients such as Hydroquinone, Vitamin C, retinoic acid, and topical steroids. They are available in various formulations, primarily including emulsions, creams, lotions, serums, and gels, all of which work through different pathways that primarily involve the inhibition of the production of melanin and skin exfoliation [5, 6].

Inappropriate use of SLP can be linked to side effects such as skin irritation, photosensitivity, skin atrophy, hypertrichosis, exogenous ochronosis, and sometimes serious complications related to mercury poisoning, like nephrotoxicity [7].

Multiple studies discussed the public health concerns associated with skin‐lightening practices among women in African and Asian countries, where lighter skin most of the time is linked to beauty and success, which can lead to psychological problems such as low self‐esteem and body image disorders. Besides, many of these products contain harmful ingredients like mercury and hydroquinone, which can lead to skin damage and other health complications [8, 9, 10]. Studies examining attitudes and perceptions of SLPs among women in medical fields are limited.

This cross‐sectional study explores the level of awareness and perceptions toward SLPs among female medical students in Jordan. As future healthcare workers, medical students have a significant role not just in patient education, but they can also influence future public health policies regarding skincare and cosmetic product safety. By investigating their understanding of these products' active components, mechanisms of action, and potential side effects, this research plans to detect the factors that affect their use and the impact of cultural beauty standards on these behaviors. The study also aims to gain insight into participants’ perceptions of lighter skin tone and its cultural significance.

Moreover, this research hopes to identify areas in professional training that need improving and tries to assist curriculum development, to help deliver more accurate counseling on these cosmetic products, in addition to contributing to wider efforts to support safe skincare habits while encouraging people to question harmful cultural beauty standards.

## 2 Materials and Methods

### 2.1 Study Design, Settings, and Participants

This descriptive cross‐sectional questionnaire‐based study, targeting all female medical students in Jordanian Universities study was conducted between (June 1, 2024) and (October 31, 2024). Female medical students currently registered at Jordanian universities were invited to complete an online survey. The survey link was distributed and shared through social media platforms, including WhatsApp and Facebook. Participation was voluntary, and students were informed that their responses would be kept confidential and anonymous. Also, they were told that the completion of the survey took approximately 10 min. After that, participants who wished to continue were asked to provide their electronic informed consent by clicking on the agree button, and for those who refused to participate, the disagree button, and no further data was collected from them.

### 2.2 Survey Instrument Development and Validation

The survey was developed based on findings from a thorough literature review, and a group of dermatologists then reviewed it to verify its accuracy and reliability; their feedback was combined, and revisions were made to enhance clarity and relevance. The questionnaire was initially tested with 20 female medical students to assess its clarity, readability, and relevance to the intended audience. To evaluate the internal consistency of the survey, Cronbach's alpha was calculated, which also helped confirm the reliability of the instrument.

The final survey consisted of four sections with a mix of closed‐ended, multiple‐choice questions and 5‐point Likert scale items. The first section is composed of participants' sociodemographic data (e.g., age and academic year). The second section evaluated the perceptions toward lighter skin tone, using a 5‐point Likert scale that ranged from strongly agree to strongly disagree. The third section evaluates their knowledge of common active ingredients in SLPs and the possible side effects associated with these components. Participants were asked to identify the active ingredients in the SLPs they used based on their knowledge, product packaging (where available), or brand familiarity. No laboratory testing was performed to verify ingredient content.

The final section explored the participants' practices, experiences, and frequency of skin‐lightening product use.

The design of the perception‐related survey items was conceptually informed by the validated instrument used by Hamed et al., which explored social perceptions of lighter skin tone among Jordanian women. Our instrument additionally included updated constructs relevant to contemporary influences such as social media and digital aesthetics [11].

### 2.3 Sample Size

The minimum representative sample size was calculated by using the Epi Info software, with a 95% confidence level, an expected frequency of 50%, and a margin of error of 5%. Based on these parameters, a sample size of 305 female medical students was required to do this study [12].

### 2.4 Ethical Considerations

This study was approved by the Ethics Committee at Jordan University of Science and Technology (IRB No. 15/168/2024). Participation was voluntary, and informed consent was obtained electronically from all participants. Respondents were informed they could withdraw at any time without consequence. No personal identifiers were collected, and all data were anonymized and used solely for research purposes.

### 2.5 Statistical Analysis

The collected data were coded and exported into a database using the Statistical Package for the Social Sciences (SPSS) version 24.0 (IBM Corp., Armonk, New York, USA). The descriptive statistics were calculated, with qualitative variables presented as frequencies and percentages. Also, continuous variables were presented as means and standard deviations.

## 3 Results

### 3.1 Characteristics of Study Participants

Our study included a total of 305 female medical students. The majority were between 20 and 23 years of age (*n* = 222, 72.8%). Most participants were in their second and fourth academic years, indicating a high representation of students in the middle stages of their studies (40.0% and 32.5%, respectively). Smoking was uncommon, with most participants identifying as non‐smokers (*n* = 276, 90.5%). Academically, the majority of students reported strong performance, with a large portion achieving either very good or excellent rankings. In terms of living arrangements, the vast majority resided at home with their families (*n* = 282, 92.5%), while a small percentage lived in hostels (*n* = 23, 7.5%). Characteristics of study participants are presented in Table 1.

**TABLE 1:** Characteristics of study participants.

	Count	Percentage
Age		
< 20	52	17.0%
20–23	222	72.8%
24–27	25	8.2%
28 or more	6	2.0%
Academic year		
First year	15	4.9%
Second year	122	40.0%
Third year	21	6.9%
Fourth year	99	32.5%
Fifth year	23	7.5%
Sixth year	25	8.2%
Smoking status		
Non‐smoker	276	90.5%
Smoker	21	6.9%
Ex‐smoker	8	2.6%
Average academic rank		
Good	78	25.6%
Very good	106	34.8%
Excellent	117	38.4%
Weak	4	1.3%
Accommodation		
Living at home	282	92.5%
Living in a hostel	23	7.5%

### 3.2 Perceptions Toward Lighter Skin Tone

The participants' perceptions of lighter skin tones were varied. Approximately 31.8% agreed or strongly agreed with the statement that lighter skin is more attractive, while 29.5% remained neutral. When asked whether a lighter skin tone gives more confidence, 45.3% disagreed, while only 28.9% agreed or strongly agreed. A notable proportion of participants (38.0%) believed that women with lighter skin have a better chance of getting married, while 40.0% disagreed or strongly disagreed.

Regarding job opportunities, 46.9% disagreed that lighter skin enhances women's employment prospects, while only 25.9% agreed. Social media's influence was evident, with 75.4% agreeing that it has led to an increase in the use of SLPs, and 78.7% agreeing that editing tools and filters reinforce lighter skin as the beauty standard. Similarly, 85.9% of participants agreed that influencers and celebrities on social media have a strong impact on beauty trends; most of the time, they promote lighter skin. Lastly, 54.1% of respondents agreed that promoting skincare and makeup products reinforces the belief that lighter skin is linked to beauty and success. Perceptions toward lighter skin tones are presented in Table 2.

**TABLE 2:** Perception of study participants toward lighter skin tone.

	Strongly disagree		Disagree		Neutral		Agree		Strongly agree	
	Count	*N* %	Count	*N* %	Count	*N* %	Count	*N* %	Count	*N* %
A lighter skin tone is more attractive	41	13.4%	77	25.2%	90	29.5%	77	25.2%	20	6.6%
Lighter skin tone gives more confidence	42	13.8%	96	31.5%	79	25.9%	71	23.3%	17	5.6%
Women with lighter skin have a higher chance of getting married	43	14.1%	79	25.9%	67	22.0%	98	32.1%	18	5.9%
Lighter skin tone increases women's job opportunities	53	17.4%	90	29.5%	83	27.2%	72	23.6%	7	2.3%
Social media has led to an increase in the use of SLPs	8	2.6%	32	10.5%	35	11.5%	145	47.5%	85	27.9%
Editing tools and filters reinforce lighter skin as the beauty standard	7	2.3%	14	4.6%	44	14.4%	130	42.6%	110	36.1%
Influencers and celebrities on social media have an impact on influencing the evolution of beauty trends and social expectations.	5	1.6%	16	5.2%	22	7.2%	143	46.9%	119	39.0%
Promoting skincare routines, makeup products, and aesthetic treatments may reinforce the belief that having a lighter skin tone is necessary for beauty and success	12	3.9%	68	22.3%	60	19.7%	119	39.0%	46	15.1%

### 3.3 Knowledge of Using Skin‐Lightening Products

Participants exhibited good knowledge regarding SLPs. A majority (62.6%) agreed that these products contain active ingredients like hydroquinone and kojic acid that inhibit melanin production, while 32.1% remained neutral. Additionally, 55.4% agreed that combining SLPs with other treatments enhances their effectiveness. Most respondents (70.2%) acknowledged that the effectiveness of these products depends on the concentration of active ingredients, though higher concentrations may increase the risk of side effects.

Regarding the regulation of SLPs, more than half (51.5%) agreed that their use should be restricted to medical purposes, while 31.6% disagreed. Moreover, 57% agreed that Hydroquinone‐containing products should not be sold over the counter, with 34.8% neutral. The majority (79%) supported the importance of using sunscreen to maintain the effectiveness of skin‐lightening treatments, and 66.2% were aware of the harmful effects of mercury in some products. Lastly, nearly two‐thirds (71.8%) agreed that steroids in SLPs can cause adverse side effects such as skin thinning, striae, rosacea, perioral dermatitis, acne, and hypertrichosis, which indicates our participants' awareness of potential health risks. Knowledge of participants about using SLPs is presented in Table 3.

**TABLE 3:** Knowledge of participants about using skin‐lightening products.

	Strongly disagree		Disagree		Neutral		Agree		Strongly agree	
	Count	*N* %	Count	*N* %	Count	*N* %	Count	*N* %	Count	*N* %
The use of skin‐lightening products should be restricted to medical purposes	8	2.6%	72	23.6%	68	22.3%	114	37.4%	43	14.1%
Skin‐lightening products may contain various active ingredients such as (Hydroquinone, kojic acid, alpha hydroxy acids, beta hydroxy acids, arbutin, and vitamin C) known for their ability to inhibit melanin production and promote the shedding of pigmented skin cells.	5	1.6%	11	3.6%	98	32.1%	157	51.5%	34	11.1%
Combining skin‐lightening products with other skincare treatments, such as exfoliants, moisturizers, or sunscreen, can enhance their effectiveness and improve overall skin health	11	3.6%	20	6.6%	105	34.4%	140	45.9%	29	9.5%
The effectiveness of SLPs can be influenced by the formulation and concentration of active ingredients. Higher concentrations are more potent but may also increase the risk of side effects such as skin irritation	5	1.6%	11	3.6%	75	24.6%	161	52.8%	53	17.4%
SLPs containing Hydroquinone should not be approved for over‐the‐counter sale.	1	0.3%	24	7.9%	106	34.8%	130	42.6%	44	14.4%
Regular use of sunscreen helps to maintain the effectiveness of SLPs and prevent the exacerbation of hyperpigmentation.	4	1.3%	8	2.6%	52	17.0%	152	49.8%	89	29.2%
SLPs containing mercury have serious side effects (Mercury is a highly toxic metal that can damage the nervous, digestive, and immune systems, as well as the lungs, kidneys, skin, and eyes)	4	1.3%	10	3.3%	89	29.2%	148	48.5%	54	17.7%
It's important to check with the doctor before using a product with Hydroquinone, as it may cause unwanted and untreatable skin discoloration (ochronosis)	6	2.0%	3	1.0%	63	20.7%	155	50.8%	78	25.6%
Steroids in some SLPs can cause skin thinning, striae, rosacea, perioral dermatitis, acne, and hypertrichosis	5	1.6%	6	2.0%	75	24.6%	161	52.8%	58	19.0%

### 3.4 Use of Skin‐Lightening Products

Of the 305 participants, 124 (40.7%) reported using SLPs; the majority of them were of Fitzpatrick skin type 3. Among those who used SLPs, the most common indications for using these products were to treat facial hyperpigmentation (35.5%) or to lighten skin tone on the face without hyperpigmentation (30.6%). The face was the most commonly treated area (73.4%), while other sites like the armpits, elbows, and knees were treated less frequently.

The majority of the students 82.3% use sunscreen in conjunction with SLP, 42.7% used them once daily at night for less than 3 months, in 43.5% of the cases SLPs were predominantly recommended by medical professionals: dermatologists, or physicians, while others relied on pharmacists (10.5%), friends (16.9%), or social media (22.6%) (Table 4).

**TABLE 4:** Use of skin‐lightening products among female medical students.

	Count	*N* %
What is your Fitzpatrick skin type		
Skin type 1	14	11.3%
Skin type 2	43	34.7%
Skin type 3	48	38.7%
Skin type 4	16	12.9%
Skin type 5	2	1.6%
Skin type 6	1	0.8%
Indication for using skin‐lightening products		
As a treatment for hyperpigmentation on the face, such as melasma	44	35.5%
For a lighter skin tone over the face with no hyperpigmentation	38	30.6%
As a treatment for hyperpigmentation on the body	36	29.0%
For lighter skin tone over particular site of the body	6	4.8%
Areas of your body did you use the skin‐lightening products on		
Face	91	73.4%
Armpits	6	4.8%
Elbows	7	5.6%
Knees	10	8.1%
Pubic and genital area	6	4.8%
Other areas	4	3.2%
Using the skin‐lightening product was based on advice from		
Physicians/Dermatologist	54	43.5%
Pharmacist	13	10.5%
Friend	21	16.9%
Social media	28	22.6%
Others	8	6.5%
Duration of using the skin‐lightening products		
Less than 3 months	53	42.7%
3–6 months	43	34.7%
6–12 months	14	11.3%
More than one year	14	11.3%
Frequency of application of skin‐lightening products		
Once daily in the morning	16	12.9%
Once daily at night	53	42.7%
Twice daily	21	16.9%
Every other day	12	9.7%
Twice ‐ three times weekly	22	17.7%
Did you use sun cream while using the skin‐lightening products		
Yes	102	82.3%
No	22	17.7%
Have you reached a satisfactory result after using the skin‐lightening products		
Yes	43	34.7%
No	35	28.2%
Partial improvement	46	37.1%

**FIGURE 1:**
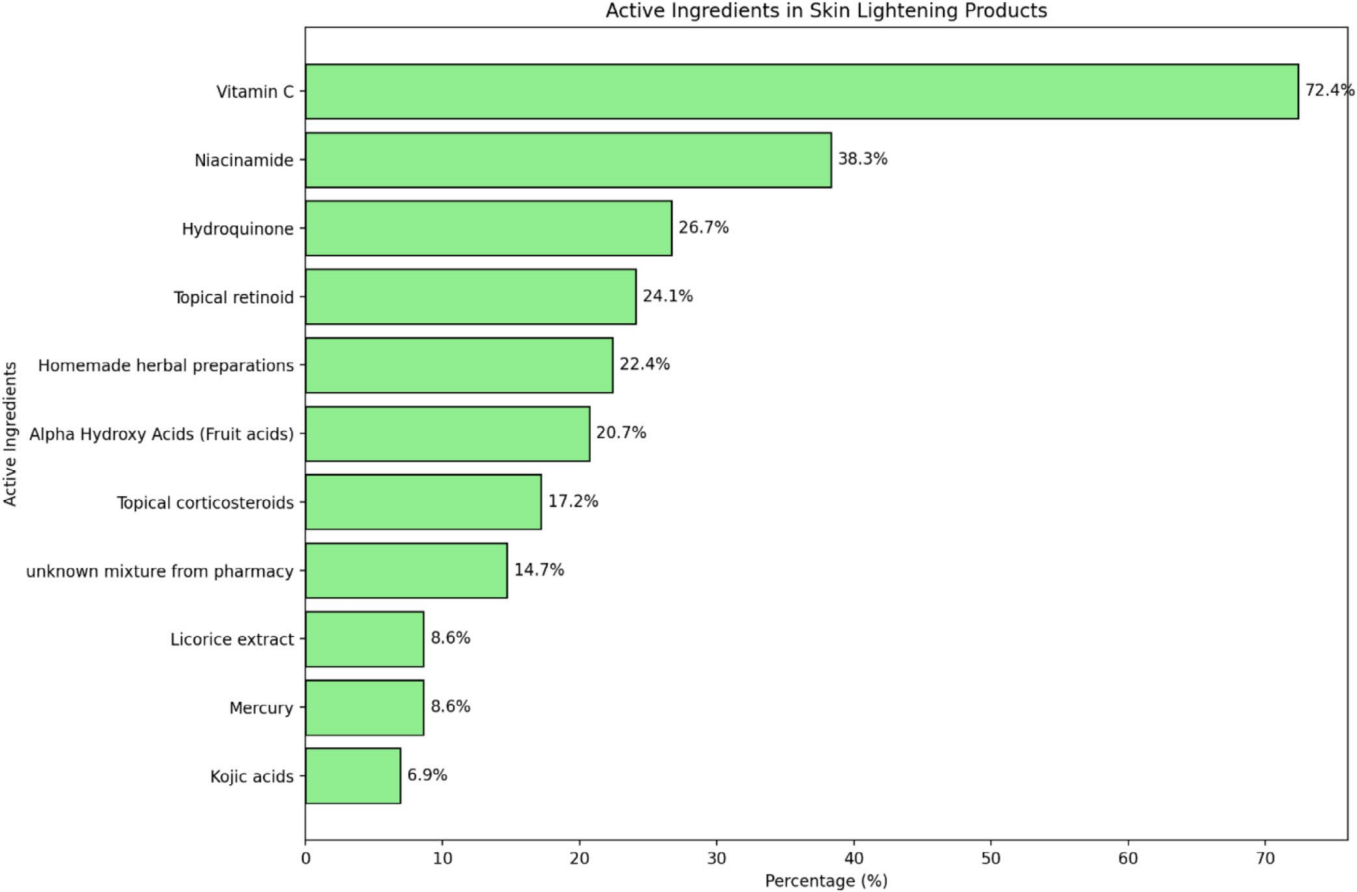
Active ingredients in the SLPs.

The most commonly reported active ingredients were Vitamin C (72.4%) and Niacinamide (38.3%). Other frequently mentioned substances included Hydroquinone (26.7%) and homemade herbal preparations (22.4%). A smaller proportion of participants reported using products they believed contained licorice extract and mercury (8.6% each) (Figure 1).

**FIGURE 2:**
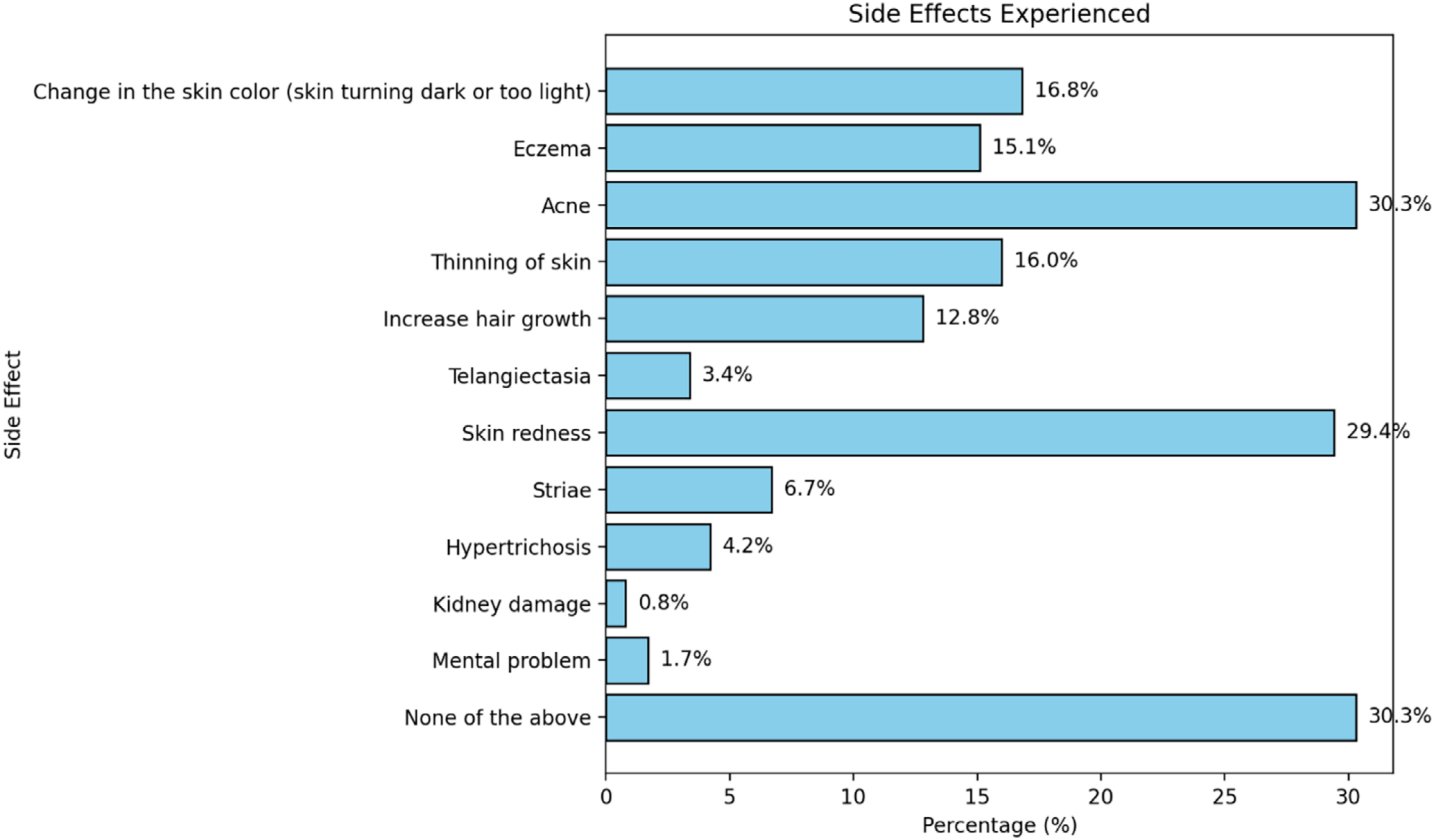
Side effects of SLPs.

Regarding side effects, 30.3% reported acne, while other effects included skin redness (29.4%) and changes in skin color (16.8%) (Figure 2).

Despite these side effects, 34.7% of users were satisfied with the results, while 37.1% reported partial improvement, and 28.2% were dissatisfied.

## 4 Discussion

The findings revealed that a significant portion of female medical undergraduates in Jordan continued to use SLPs despite possessing comprehensive knowledge about their ingredients and associated health risks. This behavior pattern reflects broader societal and cultural preferences for lighter skin tones prevalent in the region.

### 4.1 Perception of Skin Color Tone

Contrary to the findings from 2 previous national studies from Jordan that found (62.3%–70%) had a perspective that a lighter skin tone is more beautiful, in addition, 58.9% of participants believed that having a lighter skin tone improves a woman's chances of getting employed [11, 13], our results showed that 31.8% of our participants think that a lighter skin tone is more attractive and only 25.9% agreed that a lighter skin tone increases women's job opportunities.

Furthermore, our results showed that 37% believe that women with lighter skin have more chance of getting married, unlike a similar study conducted in Saudi Arabia, where more than half of the respondents (58.7%) agreed or strongly agreed that a lighter skin tone increases a woman's chances of getting married [14].

The considerable difference between our findings and the above‐mentioned previous studies regarding the perception of lighter skin tone may be attributed to the specific characteristics of our sample population—medical students—which suggests that scientific education may have a potential role in transforming beauty perspectives within society; also, it is crucial to consider the educational context when studying beauty standards and colorism.

Digital media has a crucial role in changing beauty standards by encouraging unrealistic ideals through filtered and edited images. The association between social media use and the behavior of using SLPs was evident and discussed in a recent study by Jin and Le highlighted that in Chinese society where lighter skin is often perceived as a beauty standard, the desire for social media attention can lead to increased usage of SLPs [15].

Another study from Saudi Arabia indicated that exposure to social media advertisements, beauty, and fashion television programs, and the desire to improve selfies significantly increases the interest in individuals seeking cosmetic procedures to achieve the flawless appearance they see online [16].

Our findings were consistent with previous research showing that most participants agreed on the effect of social media platforms, more than two‐thirds of them (75.4%) acknowledged that social media has increased the use of SLPs as well as the role of filters, editing tools, digital manipulation on social media, and social media celebrities in reinforcing unrealistic standards of beauty.

### 4.2 Knowledge and Attitude of Using Skin‐Lightening Products

The results show that our participants demonstrated a good understanding of the active ingredients of the SLPs, specifically recognizing the potential health risks associated with mercury. A systematic review by Bastiansz et al. analyzed data from 787 skin‐lightening product samples and found that users are at risk of variable and often high exposures to mercury, which impacts human health, affecting the nervous, cardiovascular, and immune systems, among other health concerns [17]. Their knowledge extends to hydroquinone‐related complications, such as irritant and allergic contact dermatitis, in addition to exogenous ochronosis, a condition characterized by a bluish‐black skin discoloration [18, 19]. Concerning topical corticosteroids in SLPs, students know their severe adverse effects, including epidermal atrophy, telangiectasia, and striae formation [20, 21, 22].

Despite their knowledge of the active ingredients of the SLPs and the understanding of the potential side effects associated with their misuse, a significant proportion, 40.7%, reported using SLPs, aligning with previous research among female undergraduate medical students in Nigeria that documented similar usage rates of 40.9% [23].

On the other hand, two major international studies examined skin‐lightening product use among university students. The first global study assessed skin‐lightening practices across 26 countries and found a prevalence rate of 30% among female university students. Similarly, the second research conducted across five Asian countries also documented a 30% usage rate among university students [24, 25]. These consistent findings from large‐scale international studies establish a clear baseline for general university student populations.

The notable difference in medical students' usage rates of 40.9% with the general university population of 30% reveals a significant variation in skin‐lightening practices. Despite medical students' deep understanding of product formulations and their potential risks, their usage rate exceeds the general student population by nearly 11%. This difference suggests that specialized healthcare knowledge may not be the primary influence on SLPs use; instead, factors such as peer pressure can significantly contribute to the use of SLPs, as a significant number of our participants report using these products based on recommendations from friends or social media influencers, highlighting the role of online platforms in shaping personal choices. A study from Ethiopia supports this observation; it found that a significant percentage of respondents (39.9%) indicated that peer pressure from friends influenced their decision to use SLPs [26].

Additionally, the affordability and easy accessibility, particularly through online retail platforms, of these products has markedly facilitated their use, with an extensive range of SLPs available for consumers online. A comprehensive study by Cheng et al. examining Amazon.com revealed an extensive marketplace with over 2900 skin‐lightening product listings, with consumer preference for products containing certain active ingredients known for their skin‐lightening effects, such as Vitamin E, Vitamin C, and niacinamide [27].

The commonest active ingredients used by our participants were Vitamin C (72.4%) and niacinamide (38.3%); both are widely recognized skincare ingredients easily obtainable through online retail platforms and easily incorporated into color cosmetics, reflecting a growing trend toward multifunctional beauty products.

Finally, due to society's pressure, female students sometimes focus on immediate aesthetic benefits and ignore potential long‐term health complications. Pressures from cultures significantly influence the use of SLPs, particularly in Jordan, where lighter skin is usually linked in some areas with several social advantages, including higher social status, beauty, and success.

This combination of peer pressure, easy accessibility, and cultural influence creates an environment that encourages the use of SLPs among female medical students. These findings challenge the assumption that higher levels of healthcare education automatically lead to more cautious cosmetic choices, highlighting how personal beauty practices often extend beyond professional knowledge.

## 5 Limitation

While the sample size met the precalculated threshold for statistical significance and is appropriate for this exploratory study, it may not fully capture the diversity of all female medical students in Jordan. Notably, the target population consisted exclusively of female students, and thus, the total eligible cohort was smaller than the overall national medical student population. Additionally, although the survey was distributed nationally to reach students from all Jordanian universities offering medical degrees, data on institutional affiliation or regional background were not collected to maintain participant anonymity. Future research should consider larger, multi‐institutional samples and include geographic and institutional variables to allow for subgroup analysis and improve the generalizability of findings across cultural and regional contexts.

Reported active ingredients, including mercury, were based solely on participant self‐report and not confirmed by chemical analysis. This may lead to inaccuracies due to misidentification, especially for unregulated or imported products with unclear labeling.

## 6 Conclusions

This study revealed a notable gap between knowledge and behavior. Despite participants' awareness of the risks associated with SLPs and lower endorsement of colorist beliefs, 40.7% reported using these products. Compared with the global average of 30% among female university students, this prevalence is relatively high, particularly within a medically informed population, and highlights the enduring influence of sociocultural and peer‐driven factors.

The finding highlights the need for targeted educational interventions that not only focus on scientific information but also address the underlying socio‐psychological motivations driving the use of SLPs, in addition to effective strategies that challenge common beauty standards and promote positive self‐esteem among young healthcare professionals.

Future research should focus on investigating the complex psychological factors that contribute to the knowledge‐practice gap and on developing strategic communication approaches to reduce potentially harmful cosmetic practices in healthcare settings.

## Ethics Statement

This study was approved by the Ethics Committee at Jordan University of Science and Technology (IRB No. 15/168/2024). Participation was entirely voluntary, and participants were informed of their right to withdraw at any time without consequence. No personal information was collected, and all data were fully anonymized to ensure confidentiality. The collected data were used exclusively for research purposes.

## Conflicts of Interest

The authors declare no conflicts of interest.

## Data Availability

The data that support the findings of this study are available on request from the corresponding author. The data are not publicly available due to privacy or ethical restrictions.

## References

[1] M. Dobosz , J. Radziwon , and W. J. Cubała , “Worldwide Internet Trends in the Public Interest Related to Skin Whitening and Bleaching Creams,” Journal of Cosmetic and Laser Therapy 26, no. 1–4 (2024): 26–30. 38879806 10.1080/14764172.2024.2367456

[2] S. F. Alrayyes , S. F. Alrayyes , and U. D. Farooq , “Skin‐Lightening Patterns Among Female Students: A Cross‐Sectional Study in Saudi Arabia,” International Journal of Women's Dermatology 5, no. 4 (2019): 246–250. 10.1016/j.ijwd.2019.04.026 PMC6831762 31700980

[3] Z. J. G. Regencia , J. P. Gouin , M. A. J. Ladia , J. C. Montoya , and E. S. Baja , “Effect of Body Image Perception and Skin‐Lightening Practices on Mental Health of Filipino Emerging Adults: A Mixed‐Methods Approach Protocol,” BMJ Open 13, no. 5 (2023): e068561. 10.1136/bmjopen-2022-068561 PMC10193063 37192806

[4] D. S. Saade , M. B. C. Maymone , H. De La Garza , E. A. Secemsky , K. F. Kennedy , and N. A. Vashi , “Trends in Use of Prescription Skin Lightening Creams,” International Journal of Environmental Research and Public Health 18, no. 11 (2021): 5650. 34070485 10.3390/ijerph18115650 PMC8197474

[5] N. Masub and A. Khachemoune , “Cosmetic Skin Lightening Use and Side Effects,” Journal of Dermatological Treatment 33, no. 3 (2022): 1287–1292. 33135510 10.1080/09546634.2020.1845597

[6] W. Zhao , A. Yang , J. Wang , et al., “Potential Application of Natural Bioactive Compounds as Skin‐Whitening Agents: A Review,” Journal of Cosmetic Dermatology 21, no. 12 (2022): 6669–6687. 36204978 10.1111/jocd.15437

[7] Y. M. Olumide , A. O. Akinkugbe , D. Altraide , et al., “Complications of Chronic Use of Skin Lightening Cosmetics,” International Journal of Dermatology 47, no. 4 (2008): 344–353. 18377596 10.1111/j.1365-4632.2008.02719.x

[8] S. Pollock , S. Taylor , O. Oyerinde , et al., “The Dark Side of Skin Lightening: An International Collaboration and Review of a Public Health Issue Affecting Dermatology,” International Journal of Women's Dermatology 7, no. 2 (2021): 158–164. 10.1016/j.ijwd.2020.09.006 PMC8072511 33937483

[9] J. I. Adetoogun , N. Aderinto , A. A. Ashimi , D. F. Akano , T. O. Ogundipe , and P. B. Fikayomi , “Practice and Motivations for Skin Bleaching Among Africans,” International Journal of Surgery 109, no. 2 (2023): 218–219. 36799859 10.1097/JS9.0000000000000141 PMC10389539

[10] S. Z. Rusmadi , S. N. Syed Ismail , and S. M. Praveena , “Preliminary Study on the Skin Lightening Practice and Health Symptoms Among Female Students in Malaysia,” Journal of Environmental and Public Health 2015 (2015): 1–6. 10.1155/2015/591790 PMC4674599 26693230

[11] S. H. Hamed , R. Tayyem , N. Nimer , and H. S. AlKhatib , “Skin‐Lightening Practice Among Women Living in Jordan: Prevalence, Determinants, and Users' Awareness,” International Journal of Dermatology 49, no. 4 (2010): 414–420. 20465697 10.1111/j.1365-4632.2010.04463.x

[12] Centers for Disease Control and Prevention (CDC) . “Epi Info User Guide – Chapter 12: StatCalc. US Department of Health and Human Services,” (2018), accessed June 4, 2025, https://www.cdc.gov/epiinfo/pdfs/userguide/12_statcalc.pdf.

[13] M. Ayyash , K. Jaber , R. I. Nassar , L. Fino , L. Mango , and A. Abuodeh , “Skin‐Lightening Products and Jordanian Women: Beliefs and Practice. A Cross‐Sectional Study,” PLoS One 18, no. 11 (2023): e0293896. 37988353 10.1371/journal.pone.0293896 PMC10662732

[14] S. Bamerdah , O. S. Alhothali , B. M. Aldajani , L. Alghanemi , and N. T. Mleeh , “A Cross‐Sectional Study of the Knowledge, Practice, and Attitude Towards Skin‐Lightening Products Among the General Population in the Western Region of Saudi Arabia,” Cureus [Internet] 15, no. 1 (2023): e34069, https://www.cureus.com/articles/133907‐a‐cross‐sectional‐study‐of‐the‐knowledge‐practice‐and‐attitude‐towards‐skin‐lightening‐products‐among‐the‐general‐population‐in‐the‐western‐region‐of‐saudi‐arabia. 36843720 10.7759/cureus.34069 PMC9946903

[15] R. Jin and T. T. Le , “Eyes on Me: How Social Media Use Is Associated With Urban Chinese Adolescents' Concerns About Their Physical Appearance,” Frontiers in Public Health 12 (2024): 1445090. 39145157 10.3389/fpubh.2024.1445090 PMC11322136

[16] W. S. Taishan , M. A. Ali , I. Al Sulaiman , et al., “The Effect and Implications of Social Media Platforms on Cosmetic Facial Plastic Surgery Among Females in Saudi Arabia.” (2024), Cureus [Internet], https://www.cureus.com/articles/251482‐the‐effect‐and‐implication‐of‐social‐media‐platforms‐on‐cosmetic‐facial‐plastic‐surgery‐among‐females‐in‐saudi‐arabia. 10.7759/cureus.60137 PMC11166013 38864039

[17] A. Bastiansz , J. Ewald , V. Rodríguez Saldaña , A. Santa‐Rios , and N. Basu , “A Systematic Review of Mercury Exposures From Skin‐Lightening Products,” Environmental Health Perspectives 130, no. 11 (2022): 116002. 36367779 10.1289/EHP10808 PMC9651181

[18] G. H. Findlay and H. A. de Beer , “Chronic Hydroquinone Poisoning of the Skin From Skin‐Lightening Cosmetics. A South African Epidemic of Ochronosis of the Face in Dark‐Skinned Individuals,” South African Medical Journal 57, no. 6 (1980): 187–190. 7361208

[19] O. Dadzie and A. Petit , “Skin Bleaching: Highlighting the Misuse of Cutaneous Depigmenting Agents,” Journal of the European Academy of Dermatology and Venereology 23, no. 7 (2009): 741–750. 19470077 10.1111/j.1468-3083.2009.03150.x

[20] E. Nnoruka and O. Okoye , “Topical Steroid Abuse: Its Use as a Depigmenting Agent,” Journal of the National Medical Association 98, no. 6 (2006): 934–939. 16775916 PMC2569367

[21] A. Sinha , S. Kar , N. Yadav , and B. Madke , “Prevalence of Topical Steroid Misuse Among Rural Masses,” Indian Journal of Dermatology 61, no. 1 (2016): 119. 10.4103/0019-5154.174081 PMC4763634 26955124

[22] A. S. T. Lee , N. J. Perera , and E. L. Chua , “Hypoadrenalism Secondary to Topical Corticosteroid‐Containing Skin‐Lightening Cream: Danger of Over‐The‐Counter Cosmetic Agents,” Medical Journal of Australia 203, no. 7 (2015): 287. 26424061 10.5694/mja15.00413

[23] O. G. Egbi and B. Kasia , “Prevalence, Determinants and Perception of Use of Skin Lightening Products Among Female Medical Undergraduates in Nigeria,” Skin Health and Disease 1, no. 3 (2021): e46. 35663132 10.1002/ski2.46 PMC9060047

[24] K. Peltzer , S. Pengpid , and C. James , “The Globalization of Whitening: Prevalence of Skin Lighteners (Or Bleachers) Use and Its Social Correlates Among University Students in 26 Countries,” International Journal of Dermatology 55, no. 2 (2016): 165–172. 26472662 10.1111/ijd.12860

[25] K. Peltzer and S. Pengpid , “Knowledge About, Attitude Toward, and Practice of Skin Lightening Products Use and Its Social Correlates Among University Students in Five Association of Southeast Asian Nations (ASEAN) Countries,” International Journal of Dermatology 56, no. 3 (2017): 277–283. 28093729 10.1111/ijd.13518

[26] A. T. Yayehrad , A. Lule , A. T. Tebabal , et al., “Concern on Skin Lightening Product Safety: Level of Awareness and Associated Factors Among Female Users in Bahir Dar City, Ethiopia,” CCID 16 (2023): 1753–1761. 37441697 10.2147/CCID.S416460 PMC10335271

[27] A. D. Cheng , H. De La Garza , M. B. C. Maymone , V. M. Johansen , and N. A. Vashi , “Skin‐Lightening Products: Consumer Preferences and Costs.” (2021), Cureus [Internet], https://www.cureus.com/articles/67037‐skin‐lightening‐products‐consumer‐preferences‐and‐costs. 10.7759/cureus.17245 PMC8448258 34540471

